# A randomised pragmatic trial of corticosteroid optimization in severe asthma using a composite biomarker algorithm to adjust corticosteroid dose versus standard care: study protocol for a randomised trial

**DOI:** 10.1186/s13063-017-2384-7

**Published:** 2018-01-04

**Authors:** Catherine E. Hanratty, John G. Matthews, Joseph R. Arron, David F. Choy, Ian D. Pavord, P. Bradding, Christopher E. Brightling, Rekha Chaudhuri, Douglas C. Cowan, Ratko Djukanovic, Nicola Gallagher, Stephen J. Fowler, Tim C. Hardman, Tim Harrison, Cécile T. Holweg, Peter H. Howarth, James Lordan, Adel H. Mansur, Andrew Menzies-Gow, Sofia Mosesova, Robert M. Niven, Douglas S. Robinson, Dominick E. Shaw, Samantha Walker, Ashley Woodcock, Liam G. Heaney, on behalf of the RASP-UK (Refractory Asthma Stratification Programme) Consortium, I. Adcock, A. Boriello, M. Catley, K. F. Chung, R. W. Costello, S. Johnston, F. Keane, R. May, L. Pierre, D. Smith, C. Stevenson, V. Hudson, D. Supple

**Affiliations:** 1 Centre for Experimental Medicine, School of Medicine, Dentistry and Biomedical Sciences, Queen’s University, Belfast, UK; 2 Genentech Inc., South San Francisco, California USA; 3 Nuffield Department of Medicine, The University of Oxford, Oxford, UK; 4 Department of Infection, Immunity and Inflammation, Institute for Lung Health, University of Leicester, Leicester, UK; 5 Greater Glasgow Health Board, Glasgow, UK; 6 NHS Greater Glasgow and Clyde, Stobhill Hospital, Glasgow, UK; 7 Academic Unit of Clinical and Experimental Sciences, University of Southampton, Southampton, UK; 8 Division of Infection, Immunity and Respiratory Medicine, University of Manchester, Manchester, UK; 9 Niche Science & Technology Unit 26, Falstaff House, Bardolph Road, Richmond, TW9 2LH UK; 10 University of Nottingham, Nottingham, UK; 11 University of Southampton, Centre for Inflammation, Infection and Repair, Southampton, UK; 12 The Newcastle upon Tyne NHS Foundation Trust, Newcastle upon Tyne, UK; 13 University of Birmingham and Heartlands Hospital, Heart of England NHS Foundation Trust, Birmingham, UK; 14 Royal Brompton & Harefield NHS Foundation Trust, London, UK; 15 Institute of Inflammation and Repair, The University of Manchester, Manchester, UK; 16 University College Hospitals NHS Foundation Trust, London, UK; 17 Asthma, 18 Mansell Street, London, E1 8AA UK

**Keywords:** Asthma, Biomarkers, Corticosteroids, Steroid titration, T2-low, Personalized medicine

## Abstract

**Background:**

Patients with difficult-to-control asthma consume 50–60% of healthcare costs attributed to asthma and cost approximately five-times more than patients with mild stable disease. Recent evidence demonstrates that not all patients with asthma have a typical type 2 (T2)-driven eosinophilic inflammation. These asthmatics have been called ‘T2-low asthma’ and have a minimal response to corticosteroid therapy. Adjustment of corticosteroid treatment using sputum eosinophil counts from induced sputum has demonstrated reduced severe exacerbation rates and optimized corticosteroid dose. However, it has been challenging to move induced sputum into the clinical setting. There is therefore a need to examine novel algorithms to target appropriate levels of corticosteroid treatment in difficult asthma, particularly in T2-low asthmatics. This study examines whether a composite non-invasive biomarker algorithm predicts exacerbation risk in patients with asthma on high-dose inhaled corticosteroids (ICS) (± long-acting beta agonist) treatment, and evaluates the utility of this composite score to facilitate personalized biomarker-specific titration of corticosteroid therapy.

**Methods/design:**

Patients recruited to this pragmatic, multi-centre, single-blinded randomised controlled trial are randomly allocated into either a biomarker controlled treatment advisory algorithm or usual care group in a ratio of 4:1. The primary outcome measure is the proportion of patients with any reduction in ICS or oral corticosteroid dose from baseline to week 48. Secondary outcomes include the rate of protocol-defined severe exacerbations per patient per year, time to first severe exacerbation from randomisation, dose of inhaled steroid at the end of the study, cumulative dose of inhaled corticosteroid during the study, proportion of patients on oral corticosteroids at the end of the study, proportion of patients who decline to progress to oral corticosteroids despite composite biomarker score of 2, frequency of hospital admission for asthma, change in the 7-item Asthma Control Questionnaire (ACQ-7), Asthma Quality of Life Questionnaire (AQLQ), forced expiratory volume in 1 s (FEV1), exhaled nitric oxide, blood eosinophil count, and periostin levels from baseline to week 48. Blood will also be taken for whole blood gene expression; serum, plasma, and urine will be stored for validation of additional biomarkers.

**Discussion:**

Multi-centre trials present numerous logistical issues that have been addressed to ensure minimal bias and robustness of study conduct.

**Trial registration:**

ClinicalTrials.gov, NCT02717689. Registered on 16 March 2016.

**Electronic supplementary material:**

The online version of this article (doi:10.1186/s13063-017-2384-7) contains supplementary material, which is available to authorized users.

## Background

Asthma affects an estimated 300 million people worldwide with a population prevalence of approximately 15% in the UK [1]. The WHO has estimated UK disability adjusted life-years per 100,000 population for asthma to be greater than diabetes and breast cancer [2]. In subjects with mild disease, currently available treatments work well but, in 10–20% of patients with asthma, their condition is difficult to control [3, 4]. These patients consume 50–60% of the healthcare costs attributed to asthma [5, 6] and cost approximately five-times more than patients with mild stable disease [7]. This high morbidity and disproportionate use of healthcare resources reflects the considerable un-met need in this patient group, and their significance for healthcare providers.

Asthma has been traditionally ‘stratified’ on the basis of response to ‘step-wise’ incremental treatment, with inhaled corticosteroid (ICS) therapy forming the cornerstone of this approach. However, more recently, asthma has been stratified on the basis of inflammatory phenotype to better understand disease heterogeneity with a view to developing biomarkers of therapeutic response and for better targeting of both new and existing treatments. Using sputum analysis [8], and more recently whole genome expression profiling [9], it is clear that even in mild steroid-naive asthma approximately 50% of patients do not have a typical type 2 (T2)-driven eosinophilic inflammation, and are called ‘T2-low asthma’ patients. Perhaps more significantly, on the basis of the normal diagnostic criteria for asthma, this T2-low group is indistinguishable from the typical ‘T2-high’/eosinophilic group. However, in the context of therapeutic response, the T2-low patients have a minimal response to ICS therapy [8, 9]. In severe disease, the T2-low profile is also prevalent [10, 11], and in case series of difficult-to-treat patients with asthma there is substantial evidence that inappropriate escalation of corticosteroid treatment is frequent, with significant morbidity due to systemic corticosteroid exposure [12–14]. In a follow-up analysis of the largest “real-world” refractory asthma cohort to date in the British Thoracic Society Difficult Asthma Network in the UK [15] the most common therapeutic intervention in specialist centres was the addition of systemic corticosteroid treatment, with approximately 60% ending up on oral corticosteroids [16].

Given the evidence that corticosteroid responsiveness is minimal in the absence of T2-driven inflammation, there is a need to examine novel algorithms to target appropriate levels of corticosteroid treatment in difficult asthma, particularly in patients with low T2 biomarkers. Patients with high T2 biomarkers are at risk of severe exacerbations (e.g. baseline fractional exhaled nitric oxide (FeNO) > 45 ppb predicts frequent exacerbation [17]), and in this group the key clinical question is whether this is because of non-adherence to inhaled corticosteroid treatment or whether they have relative corticosteroid-resistant T2-high asthma and require additional treatment with biological drugs that target T2 mechanisms (e.g. antibodies that inhibit interleukin (IL)-5 or IL-13 signalling). Previous studies in severe asthma have shown that titrating corticosteroids against sputum eosinophilia may be efficacious without increasing cumulative ICS burden [18], but a sputum monitoring strategy has not been widely taken up in most secondary or tertiary care settings because it poses practical implications that make it difficult to deliver in the clinic [18]. In a number of studies FeNO monitoring has not shown utility in disease management [18]. Whereas improved disease outcome was demonstrated in mild asthma in pregnancy [19], the value of FeNO in managing corticosteroid adjustment in more severe disease is uncertain [15, 18, 20]. Periostin is a secreted matricellular protein associated with fibrosis whose expression is upregulated by recombinant IL-4 and IL-13 in cultured bronchial epithelial cells. Measurement of serum periostin has been shown to be superior at identifying airway eosinophilia (defined as a composite of bronchial biopsy or sputum eosinophilia) than sputum alone or FeNO alone [21], although this was not seen in another study when looking at sputum eosinophilia alone [22].

We have examined the value of a composite biomarker strategy using FeNO, blood eosinophils, and serum periostin together to predict exacerbation risk in the placebo arms of clinical trials with lebrikizumab (anti-IL-13) and omalizumab (anti-IgE) in patients taking at least 500 μg fluticasone propionate (FP) and a second asthma controller [23–25]. These studies were designed to prospectively collect exacerbation events.

This analysis has demonstrated that each of the three biomarkers (blood eosinophils, FeNO, serum periostin) are individually prognostic for exacerbation risk, and using the three biomarkers in combination through a ‘composite’ scoring system has the potential to refine this risk prediction (Table 1).

**Table 1:** The composite biomarker score is calculated from the individual biomarker scores, and is the mean of all three scores rounded to the nearest integer to give the “composite score” (score of 0, 1 or 2)

Scoring system	0	1	2
Fractional exhaled NO (ppb)	< 15	≥ 15 to < 30	≥ 30
Blood eosinophil count (N/μL)	< 150	≥ 150 to < 300	≥ 300
Periostin (ng/ml)	< 45	≥ 45 to < 55	≥ 55

We propose to examine if this composite biomarker strategy predicts exacerbation risk in patients with asthma on high-dose ICS (± long-acting beta agonist (LABA)) treatment and to evaluate the utility of this composite score to facilitate personalised biomarker-specific titration of corticosteroid therapy in this population. This study will examine subjects with FeNO < 45 ppb, and the scoring system will potentially allow identification of a ‘low-risk’, corticosteroid-unresponsive group in which we can safely reduce corticosteroid dose. This study will address a second important question of estimating the proportion of patients with severe disease who develop typical (T2)-driven eosinophilic inflammation on progressive corticosteroid withdrawal.

Our hypothesis is that adjusting corticosteroid therapy using composite biomarker scores will lead to more appropriate corticosteroid dosing in severe asthma, with no increase in exacerbation risk and a reduction in corticosteroid load compared to standard care. Current standard care in asthma management guidelines adjusts corticosteroid treatment based on symptoms, lung function, and exacerbation history, and we will compare biomarker-based adjustment to this standard care. This is essential to confirm that a biomarker-based strategy delivers better clinical outcomes compared to standard care.

## Methods/design

This is a randomised, pragmatic, single-blind (study participant), multi-centre, controlled, parallel group trial in patients with severe asthma (Global Initiative for Asthma (GINA) steps 4 and 5 classification of asthma severity). It will compare a composite biomarker-based (blood eosinophils, periostin, or FeNO data) adjustment of corticosteroid therapy (biomarker arm, see Tables 2 and 3) versus adjustments based on asthma symptom control and lung function (standard symptom-based care). Trial staff in clinical centres where a biomarker-based treatment strategy is already used as standard of care (including a sputum-based strategy) have been instructed that patients in the symptom-based arm will have therapy adjusted according to the clinical trial algorithm to mirror international treatment guidelines and current clinical practice in the UK (Tables 4 and 5).

**Table 2:** Biomarker-based therapy adjustment

Score	Corticosteroid dose step-wise adjustment	Follow-up
0	Reduce treatment 1 step	If score remains 0 on low-dose corticosteroid – type 2 (T2)-low severe asthma
1	Maintain current treatment	Adjust as necessary based on follow-up scores
2	Increase treatment 1 step	If score remains 2 despite maximal inhaled corticosteroid therapy necessitating systemic steroid therapy – T2-high severe asthma

All therapeutic adjustments will be automatically calculated and advised by the electronic case report form (e-CRF)

Corticosteroid treatment adjustment based on composite biomarker score

**Table 3:** Treatment steps for biomarker-based therapy adjustment

Steroid therapy step	Seretide MDI	Seretide Accuhaler	Symbicort Turbohaler	Flutiform MDI	Relvar Ellipta	Other LABA/ICS combinations (FP equivalent dose per day)
Step 1	Seretide 50 2 bd	Seretide 100 1 bd	Symbicort 6/200 1 bd If ACQ ≥ 1.5 add OXIS 12 1 bd (or equivalent)	Flutiform 50 2 bd If ACQ ≥ 1.5 add OXIS 12 1 bd (or equivalent)	Seretide 100 Accuhaler 1 bd	LABA/FP equivalent – 200 μg per day
Step 2	Seretide 125 2 bd	Seretide 250 1 bd	Symbicort 6/200 2 bd If ACQ ≥ 1.5 add OXIS 12 1 bd (or equivalent)	Flutiform 125 2 bd If ACQ ≥ 1.5 add OXIS 12 1 bd (or equivalent)	Relvar 22/92 1 mane	LABA/FP equivalent – 500 μg per day
Step 3	Seretide 250 2 bd	Seretide 500 1 bd	Bud 12/400 2 bd	Flutiform 250 2 bd	Relvar 22/184 1 mane	LABA/FP equivalent – 1000 μg per day
Step 4	Seretide 250 2 bd μg Prednisolone 5 mg per day	Seretide 500 1 bd Prednisolone 5 mg per day	Bud 12/400 2 bd Prednisolone 5 mg per day	Flutiform 250 2 bd Prednisolone 5 mg per day	Relvar 22/184 1 mane Prednisolone 5 mg per day	LABA/FP equivalent – 1000 μg per day Prednisolone 5 mg per day
Step 5	Seretide 250 2 bd Prednisolone 10 mg per day	Seretide 500 1 bd Prednisolone 10 mg per day	Bud 12/400 2 bd Prednisolone 10 mg per day	Flutiform 250 2 bd Prednisolone 10 mg per day	Relvar 22/184 1 mane Prednisolone 10 mg per day	LABA/FP equivalent – 1000 μg per day Prednisolone 10 mg per day
Step 6	Seretide 250 2 bd Prednisolone 15 mg per day	Seretide 500 1 bd plus Prednisolone 15 mg per day	Bud 12/400 2 bd plus Prednisolone 15 mg per day	Flutiform 250 2 bd Prednisolone 15 mg per day	Relvar 22/184 1 mane plus Prednisolone 15 mg per day	LABA/FP equivalent – 1000 μg per day plus Prednisolone 15 mg per day
Step 7*	Seretide 250 2 bd Prednisolone 20 mg per day	Seretide 500 1 bd Prednisolone 20 mg per day	Bud 12/400 2 bd Prednisolone 20 mg per day	Flutiform 250 2 bd Prednisolone 20 mg per day	Relvar 22/184 1 mane Prednisolone 20 mg per day	LABA/FP equivalent – 1000 μg per day plus Prednisolone 20 mg per day

* It is recognised that on some occasions patients may require higher doses of systemic steroids beyond 20 mg prednisolone per day; as with all treatment steps, particular attention should be paid to adherence with prednisolone, but if required prednisolone can be increased in further 5 mg increments

The therapeutic adjustments are designed to reflect clinical practice and to be pragmatic and allow accommodation of currently used combination inhaler therapies in this population; because of this, ICS will be adjusted in line with the patient’s prescribed LABA/ICS inhaler device. This will mean in some situations that LABA is adjusted along with ICS which would reflect usual clinical practice

If patient is on theophylline, leukotriene receptor antagonist, at baseline, these are not adjusted during study; they are not added during the study

If patient has an Asthma Control Questionnaire (ACQ)7 > 1.5 and corticosteroid is *not* increased, tiotropium should be added if no contraindications if patient is not already on Long-Acting Muscarinic Antagonist (LAMA) therapy or nebulised short-acting anti-muscarinic therapy

If on inhaled steroid monotherapy (nebulised or inhaled) in addition to ICA/LABA combination therapy, the inhaled steroid monotherapy will be withdrawn initially

If a patient is on oral steroids and reduces to 5 mg per day, they should be advised to omit their prednisolone on the morning of their next study visit; at that visit, they should have a morning cortisol checked locally as part of routine clinical care: if cortisol within normal range of local laboratory reference value, steroids can be stopped completely if indicated by study algorithm; if cortisol is present but outside normal reference range of local laboratory, gradual oral steroid withdrawal in 1 mg increments is carried out; if cortisol is undetectable, prednisolone is maintained at 5 mg for study duration

*bd* twice a day, *Bud* budesonide, *FP* fluticasone propionate, *ICS* inhaled corticosteroid, *LABA* long-acting beta agonist, *mane* every morning, *MDI* metered dose inhaler

**Table 4:** Symptom-based therapy adjustment (all therapeutic adjustments will be automatically calculated and advised by the e-CRF)

Asthma Control (ACQ7)	Treatment increased according to Table 5
ACQ7 ≥ 1.5 and ≥ 1 change from baseline score *OR* severe exacerbation since last visit (past 8 weeks at baseline randomisation visit)	Increase therapy 1 step
ACQ7 is 1.0 to < 1.5 *OR* ACQ ≥ 1.5 and < 1 change from baseline score *AND* no severe exacerbation since last study visit (past 8 weeks at baseline randomisation visit)	No change
ACQ7 < 1.0 *AND* no severe exacerbation since last study visit (prior 8 weeks at baseline randomisation visit)	Reduce therapy 1 step

All therapeutic adjustments will be automatically calculated and advised by the electronic case report form (e-CRF)

*ACQ7* 7-item Asthma Control Questionnaire

**Table 5:** Treatment for symptom-based therapy adjustment (British Thoracic Society guidelines)

Step 1	LABA/low-dose ICS (FP 200 μg or equivalent)
Step 2	LABA/moderate-dose ICS (500 μg FP equivalent)
Step 3	LABA/high-dose ICS (1000 μg FP equivalent)
Step 4	Add Tiotropium
Step 5*	Add regular oral steroids (starting dose 5–10 mg per day increasing in 5 mg increments)

* It is recognised that on some occasions patients may require higher doses of systemic steroids beyond 20 mg prednisolone per day; as with all treatment steps, particular attention should be paid to adherence with prednisolone, but if required prednisolone can be increased in further 5-mg increments

The therapeutic adjustments are designed to reflect clinical practice and to be pragmatic and allow accommodation of currently used combination inhaler therapies in this population; because of this, ICS will be adjusted in line with the patient’s prescribed LABA/ICS inhaler device. This will mean in some situations that LABA is adjusted along with ICS which would reflect usual clinical practice

If patient is on theophylline, leukotriene receptor antagonist, at baseline, these are not adjusted during study; they are not added during the study

If patient has an Asthma Control Questionnaire (ACQ)7 > 1.5 and corticosteroid is *not* increased, tiotropium should be added if no contraindications if patient is not already on Long-Acting Muscarinic Antagonist (LAMA) therapy or nebulised short-acting anti-muscarinic therapy

If on inhaled steroid monotherapy (nebulised or inhaled) in addition to ICA/LABA combination therapy, the inhaled steroid monotherapy will be withdrawn initially

If a patient is on oral steroids and reduces to 5 mg per day, they should be advised to omit their prednisolone on the morning of their next study visit; at that visit, they should have a morning cortisol checked locally as part of routine clinical care: if cortisol within normal range of local laboratory reference value, steroids can be stopped completely if indicated by study algorithm; if cortisol is present but outside normal reference range of local laboratory, gradual oral steroid withdrawal in 1 mg increments is carried out; if cortisol is undetectable, prednisolone is maintained at 5 mg for study duration

*FP* fluticasone propionate, *ICS* inhaled corticosteroid, *LABA* long-acting beta agonist

The study is being conducted in 12 centres throughout England, Scotland, and Northern Ireland in accordance with the ethical principles that have their origin in the Declaration of Helsinki, ICH Good Clinical Practice (GCP), and all applicable regulations. Queens University Belfast is the principal sponsor for this study (Ref. 15062LH-AS). A SPIRIT (Standardized Protocol Items: Recommendations for Interventional Trials) checklist accompanies this protocol to verify that all items required to be addressed in a high-quality trail are included in this protocol (see Additional file 1). The protocol has been reviewed and approved by the Office for Research Ethics Northern Ireland (Ref. 15/NI0158). All individual sites have given local National Health Service Research and Development (NHS R&D) approval. The trial has been registered with ClinicalTrials.gov (NCT02717689).

### Eligibility criteria

The study is recruiting patients aged 18 to 80 years old inclusive with severe asthma (GINA steps 4 and 5 classification) who are currently attending difficult asthma clinics in specialist centres. All screened non-randomised patients have the following anonymised details recorded for CONSORT (Consolidated Standards of Reporting Trials) reporting: age; gender; ethnicity (if applicable); and the reason not eligible for trial participation, or if they are eligible but declined.

#### Inclusion criteria

Patients must meet the following criteria at screening for study entry (patients can be rescreened for study entry up to three times):

1. Age ≥ 18 and ≤ 80 years at screening visit.
2. Able and willing to provide written informed consent and to comply with the study protocol.
3. Baseline FeNO < 45 ppb at screening.
4. Severe asthma confirmed after assessment by an asthma specialist. Diagnosed with asthma at least 12 months prior to screening.
5. Current asthma treatment with LABA plus high doses of inhaled corticosteroids (≥1000 μg FP daily or equivalent).
6. Patients on an ICS/LABA single inhaler strategy must be switched to fixed dosing ICS/LABA for 4 weeks prior to screening.
7. Documented history of reversibility of ≥ 12% change in forced expiratory volume in 1 s (FEV1) within the past 24 months or during screening period, as demonstrated by:
  documented airflow obstruction (FEV1/forced vital capacity (FVC)) < 70%), where FEV1 has varied by ≥ 12% either spontaneously or in response to oral corticosteroid (OCS) therapy or bronchodilators either between or during clinic visits.
*or*
  A 20% drop in FEV1 (PC_20_) to methacholine < 8 mg/mL or a 15% fall in FEV_1_ (PD_15_) after inhaling a cumulative dose of mannitol of ≤ 635 mg indicating the presence of airway hyperresponsiveness. If sites customarily use histamine to perform tests of airway responsiveness, this may be used in place of methacholine.

#### Exclusion criteria

Patients who meet any of the following criteria are excluded from study entry:

1. Acute exacerbation requiring oral corticosteroids in previous 4 weeks before screening.
2. Known severe or clinically significant immunodeficiency, including, but not limited to, human immunodeficiency virus (HIV) infection.
3. Currently receiving or have historically received intravenous immunoglobulin for treatment for immunodeficiency.
4. If recently commenced on a leukotriene receptor antagonist or theophylline, stable on treatment for 4 weeks prior to screening.
5. Known current malignancy or current evaluation for a potential malignancy or history of malignancy within 5 years prior to baseline. With the exception of basal-cell and squamous-cell carcinomas of the skin and carcinoma in situ of the cervix uteri that have been excised and cured.
6. Other clinically significant medical disease or uncontrolled concomitant disease despite treatment that is likely, in the opinion of the investigator, to require a change in therapy or impact the ability to participate in the study.
7. History of current alcohol, drug, or chemical abuse or past abuse that would impair or risk the subject’s full participation in the study, in the opinion of the investigator.
8. Current self-reported history of smoking (including electronic inhaled nicotine products) or former smoker with a smoking history of > 15 pack-years
  - A current smoker is defined as someone who has smoked one or more cigarettes per day (or marijuana or pipe or cigar) for ≥ 30 days within the 24 months prior to the screening visit (day –14) and/or cotinine positive at screening.
  - Any individual who smokes (cigarettes, marijuana, pipe, or cigar) occasionally, even if for < 30 days within the 24 months prior to the screening visit (day –14), must agree to abstain from all smoking from the time of consent through to completion of study.
  - A former smoker is defined as someone who has smoked one or more cigarettes per day (or marijuana or pipe or cigar) for ≥ 30 days in his or her lifetime (as long as the 30-day total did not include the 24 months prior to the screening visit (day –14)).
  - A pack-year is defined as the average number of packs per day times the number of years of smoking.
9. Current use of an immunomodulatory/immunosuppressive therapy or past use within 3 months or five drug half-lives (whichever is longer) prior to the screening visit.
10. Use of a biologic therapy including omalizumab at any time during the 6 months prior to the screening visit.
11. Bronchial thermoplasty within the prior 6 months of the screening visit.
12. Initiation of or change in allergen immunotherapy within 3 months prior to the screening visit.
13. Treatment with an investigational agent within 30 days of the screening visit (or five half-lives of the investigational agent, whichever is longer).
14. Female patients who are pregnant or lactating.

### Safety

Safety is assessed by standard adverse event (AE) and serious adverse event (SAE) reporting and assessment of clinical laboratory results. In this study population it is recognised that severe asthma is a variable condition, prone to worsening and exacerbation. Therefore, for the purposes of AE reporting, asthma exacerbations are classed as expected AEs. Patient safety is monitored by documenting AEs and the procedures described below for expedited reporting. All AEs reported or observed during the study are recorded on the AE page in the electronic case report form (e-CRF). The Medical Dictionary for Regulatory Activities (MedDRA) is being used to code all AEs. SAEs suspected by the investigator to be associated with study procedures are required to be reported by the investigator to the Sponsor immediately (i.e. within 24 h after learning of the event). Given the hypothesis-generating nature of this study, the Sponsor may choose to conduct an interim efficacy analysis. The decision to conduct an interim analysis and the timing of the analysis will be documented in the statistical analysis plan prior to the conduct of the interim analysis. The interim analysis will be performed and interpreted by members of the Trial Management Group and RASP-UK Executive Management Team. Safety analyses will include all randomised patients, with patients allocated to the group associated with the regimen they actually received.

### Power and sample size estimate

The primary efficacy endpoint of this study is the proportion of patients who achieve any reduction in ICS or OCS dose from baseline to week 48. It is estimated that up to 10% of the patients in the symptom-based algorithm (control) group will achieve a reduction in ICS or OCS. Operating characteristics for achieving additional reductions in the biomarker-based algorithm (active intervention) group are provided in Table 6, with 200, 300, and 400 subjects with each scenario assuming a patient drop-out rate of 20% by week 48 with > 80% power at the two-sided α = 0.05 significance level with use of the continuity-corrected chi-squared test for proportions.

**Table 6:** Operating characteristics for detecting differences in the primary endpoint

Total N (intervention + control)	Ratio	Type 1 error	Proportion of patients achieving a reduction in ICS or OCS			Power
			Control	Intervention	Difference	
400 (320 + 80)	4:1	0.05	10%	26%	16%	83%
300 (240 + 60)	4:1	0.05	10%	29%	19%	83%
200 (160 + 40)	4:1	0.05	10%	34%	24%	83%

Assumes a 20% drop out rate

*ICS* inhaled corticosteroid, *OCS* oral corticosteroid

**Fig. 1:**
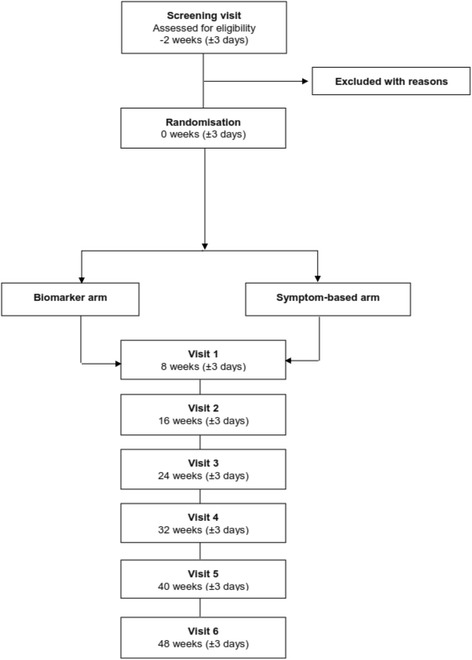
CONSORT diagram displaying flow of patients through the RASP-UK biomarker study

Efficacy analyses will be conducted on an intent-to-treat (ITT) population consisting of all randomised participants who completed at least one post-baseline efficacy assessment, with patients allocated to the group to which they were randomised. The number and proportion of patients who enrol, discontinue, and complete the study will be tabulated by the Trial Management Group. Reasons for early termination from the study will be listed and summarised by Trial Management Group. Any eligibility criteria exceptions and other major protocol deviations will also be summarized by the Trial Management Group. A full CONSORT diagram (Fig. 1) will be produced for the study including patients screened and reason for screen failure. Demographic and baseline characteristics (e.g. age, sex, ethnicity, weight, FEV1) will be summarized for all randomised patients by the Trial Management Group using descriptive statistics.

### Outcome measures and data analysis

The primary outcome measure is the proportion of patients with any reduction in ICS or OCS dose from baseline to week 48. For this endpoint, the Cochran–Mantel–Haenszel chi-square test (or Fisher’s exact) test will be used to compare the two treatment management arms with additional analysis as required. The descriptive summaries will include as a minimum counts and proportions. Secondary outcomes consist of the following:

1. Rate of protocol-defined severe exacerbations* per patient per year during the 48-week study period post-randomisation.
2. Time in days to first severe exacerbation from randomisation.
3. Dose of inhaled steroid (μg fluticasone propionate equivalent) at 48 weeks or at last visit if patient withdraws early.
4. Cumulative dose of inhaled corticosteroid during the 48-week study period.
5. Proportion of patients on oral corticosteroids at the end of the 48-week study period.
6. Proportion of patients who decline to progress to oral corticosteroids despite composite biomarker score of 2.
7. Frequency of hospital admission for asthma throughout the 48-week study period.
8. Change in the 7-item Asthma Control Questionnaire (ACQ-7) from baseline to week 48.
9. Relative change in FEV1 (volume) from baseline to week 48?
10. Change in exhaled breath nitric oxide (FeNO), blood eosinophil count and periostin levels from baseline to week 48?
11. Change in Asthma Quality of Life Questionnaire (AQLQ) from baseline to week 48.

* A severe asthma exacerbation is defined as new or increased asthma symptoms (including wheeze, cough, dyspnoea, or nocturnal awakenings due to these symptoms) that leads to: 1) at least a doubling of treatment with OCS for ≥ 3 consecutive days (for subjects on maintenance OCS); 2) an increase in treatment with OCS to the usual rescue course of oral steroids (usually ≥ 0.5 mg/kg for subjects not on maintenance OCS for ≥ 3 consecutive days); and 3) administration of intravenous and/or intramuscular corticosteroids for asthma hospitalization for asthma.

The rate of protocol-defined severe exacerbations per patient per year during the 48-week study period post-randomisation will be estimated by the total number of protocol-defined exacerbations observed in the group over the treatment period divided by total patient-weeks at risk for the group. For each individual patient, weeks at risk will be computed as the number of days between the study completion or study discontinuation date and the date of randomisation (baseline), divided by 7 days. Poisson regression with over-dispersion will be used in the analysis to assess the treatment effect on the rate of protocol-defined asthma exacerbations. The model will adjust for baseline inhaled steroid dose, asthma control (ACQ-7 ≤ 1.25 vs > 1.25), and the use of ‘rescue’ steroids during the past 12 months.

For the continuous endpoints, analysis methods such as analysis of variance (ANOVA), analysis of covariance (ANCOVA), or non-parametric tests will be implemented. The descriptive summaries will include mean, standard deviation, median, and range. For the categorical variables, the Cochran–Mantel–Haenszel χ2 (or Fisher’s exact) test will be used to compare the two treatment management groups. The descriptive summaries will include counts and proportions.

Time to first severe exacerbation from randomisation will be compared between treatment management groups using a two-sided stratified log-rank test. Results from an unstratified log-rank test will also be presented. The proportion of subjects reporting severe exacerbations at each visit will be estimated using Kaplan-Meier methodology. Estimation of the hazard ratio will be based on a stratified Cox regression model with the same stratification factor used in the stratified log-rank test above. The estimates of the unstratified hazard ratio may also be presented.

The e-CRF has been designed so that all endpoint data will have to mandatorily be completed prior to concluding each visit. If an item is not available or is not applicable (e.g. a pregnancy test in a post-menopausal woman or male), this will be indicated. Any endpoint-sensitive data is highlighted to the algorithm keeper in ‘real-time’ and queried with the clinical centre. In the event of missing data, analyses of change from baseline endpoints will use last observation carried forward (LOCF) imputation for missing values. Missing data for patient-reported outcome (PRO) assessments (e.g. ACQ-7, AQLQ) will not be imputed, with the exception of PRO instruments with specific instructions for handling missing data. Analyses of the primary endpoint and other asthma exacerbation endpoints will be based on observed data with no imputation for premature discontinuation from the study.

### Biobanking and DNA sampling

**Fig. 2:**
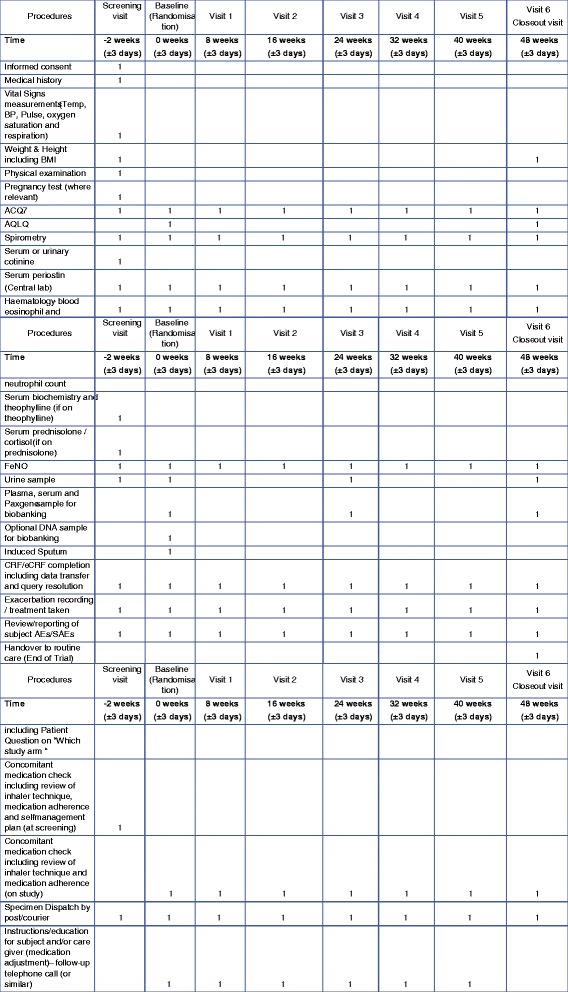
Schedule of study procedures. *ACQ-7* 7-item Asthma Control Questionnaire, *AE* adverse event, *AQLQ* Asthma Quality of Life Questionnaire; *BMI* body mass index, *BP* blood pressure, *(e-)CRF* (electronic) case report form, *FeNO* fractional exhaled nitric oxide, *SAE* serious adverse event, *Temp* temperature

At baseline and selected study visits (Fig. 2) blood is taken for whole blood gene expression. Serum, plasma, and urine will be stored for validation of additional biomarkers including, but not limited to, biomarkers related to asthmatic airway inflammation, corticosteroid signalling, and putative inflammatory pathways in severe asthma. There is also an optional whole blood sample taken for DNA extraction. This optional DNA sample is collected from patients who also sign the separate informed consent for a DNA sample to be taken and stored in the Roche Clinical Repository (RCR). The RCR is a centrally administered group of facilities for the long-term storage of human biologic specimens, including body fluids, solid tissues, and derivatives thereof (e.g. DNA, RNA, proteins, peptides). The purpose of collecting this sample is to determine genetic factors that may predict response to glucocorticoids, as well as to understand the relationship between heritable factors and response to any therapy typically used in the treatment of uncontrolled asthma, clinical features of uncontrolled asthma, or safety events. The DNA sample may also be useful in the discovery of new drug targets or to identify new biological pathways involved in asthma, developing biomarker or diagnostic assays and to establish the performance characteristics of these assays, and increasing knowledge and understanding of inflammatory diseases and their biology.

An important exploratory objective of this study is to phenotype exacerbations in both arms. All patients are asked to contact their clinical centre to arrange clinical assessment when there is deterioration in their baseline asthma control for which they plan to increase their medication according to their self-management plan. This plan is reviewed at the screening visit (it is recognised this may be outside the normal working week (Monday to Friday) during which patients are advised to follow their management plan and contact their clinical centre on the next working day or attend out of hours/emergency care if needed).

## Trial conduct

### Consent

Informed consent is obtained by the Principal Investigator (PI), or named delegate, at each site prior to the study participant undergoing procedures. A separate consent form is used for the optional RCR DNA sample (Additional file 2). Sampling for the RCR is contingent upon the review and approval of the exploratory research and the RCR portion of the ICF by each site’s NHS R&D Committee or Ethics Committee and, if applicable, an appropriate regulatory body. If a site has not been granted approval for RCR sampling, this section of the protocol will not be applicable at that site.

### Treatment allocation and randomisation

Patients are randomly allocated to either the biomarker-based arm or symptom-based standard care arm. Treatment is allocated by a centrally managed computer algorithm designed by Dendrite Clinical Systems. The ratio of allocation of patients is 4:1 biomarker-based to symptom-based. The rationale for using the differential randomisation ratio is to allow adequate capture of exacerbation events in the biomarker arm, thus enabling phenotyping of exacerbations in this group. Randomisation is also stratified by asthma control (ACQ-7 ≤ 1.5 vs > 1.5) and use of ‘rescue’ steroids during the past 12 months. Allocation to the treatment algorithm group is concealed from the participant, and investigators analysing outcomes will be masked to group assignment.

### Study procedures

Patients will undergo a screening visit (day –14), a baseline visit (day 0) and six follow-up visits at weeks 8, 16, 24, 32, and 48 (see Fig. 2 for the schedule of study procedures). FeNO will be measured using the Aerocrine Vero® [26, 27]. Periostin will be measured using the Elecsys® Periostin assay developed by Roche Diagnostics (Penzberg, Germany) [28]. Blood eosinophil count will be measured using local haematology laboratory automated counters reflecting the pragmatic clinical design.

**Fig. 3:**
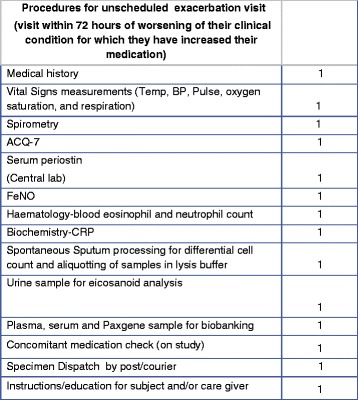
Schedule of study procedures: unscheduled exacerbation visit. *ACQ-7* 7-item Asthma Control Questionnaire, *BP* blood pressure, *CRP* C-reactive peptide, *FeNO* fractional exhaled nitric oxide, *Temp* temperature

If a patient attends for an exacerbation visit the procedures outlined in Fig. 3 will be conducted.

### Data collection

All clinical trial source documents are held at each site and all clinical trial visits are documented in the patient’s clinical records held at the clinical site in line with GCP. Trial data is being captured by a password -protected e-CRF hosted by Dendrite Clinical Systems Ltd. (http://www.e-dendrite.com/). This will provide point-of-care clinical trial data capture, which assigns a unique identifier number at the time consent is given. All data are therefore anonymised and only linked to patient-identifiable data held securely and locally at the individual NHS clinical sites. This system ensures that all data points are completed at data entry and data entry errors are minimised. Data checking and cleaning and review will be performed prior to data lock and transfer into the knowledge management system. The Health Informatics system performs automatic system back-ups in real time ensuring data cannot be lost and can always be retrieved (Dendrite Clinical Systems). The database is monitored by a full audit trail that logs all data additions, modifications, and deletions for users of the system.

### Trial oversight and monitoring

The chief investigator (LGH) maintains overall responsibility for the conduct of the trial. There is also a Trial Management Group (comprised of independent clinicians and patient representatives) and the RASP-UK Executive Management Team overseeing the setup, conduct, analysis, and dissemination of the study. Composition, roles, and responsibilities of these groups can be obtained by contacting the clinical monitors (Niche Science and Technology Ltd.). As a representative of the sponsor, Clinical Research Associates from this company visit the investigator and study facilities at periodic intervals, in addition to maintaining necessary telephone and email correspondence. The monitor maintains current personal knowledge of the study, including recruitment progress, through observation, review of study records and source documentation, and discussion of the conduct of the study with the investigator and staff. All aspects of the study are being carefully monitored by the sponsor or its designee for compliance with applicable government regulation with respect to current GCP and current standard operating procedures.

### End of trial/early patient withdrawal

The final study visit occurs 48 weeks after randomisation when all patients have a final assessment. At this visit, patients have blood samples collected for measurement of periostin levels and other on-study measurements (spirometry, ACQ-7, FeNO, PAXgene, and serum sampling). Exacerbations and any additional treatment in the final study month as well as other concurrent treatment are recorded. Subjects are also asked which treatment adjustment arm they were in during the study (biomarker versus standard care). The study will be considered complete with the last study assessment for the last patient participating in the study.

Patients will be withdrawn from the study at the discretion of the local PI or Chief Investigator (CI) should anything happen that would adversely affect their participation or clinical care or require them to have conflicting treatment, e.g. increased steroid use for inflammatory or haematological conditions.

## Discussion

As a multi-centre trial, there have been numerous logistical issues that have been addressed to ensure minimal bias and quality and robustness of study conduct. This study has been designed to be pragmatic and therefore more reflective of routine clinical management of patients with severe asthma, and this will enable the results to be more clinically relevant and applicable. In developing this protocol, the RASP-UK Consortium formed a public and patient representative group to gain input from patient representatives. Furthermore, patient representatives have been involved in ongoing review of participation and site issues.

The pragmatic nature of this study necessitated a single-blind study design. In a normal clinical setting, patients who are managed according to their symptoms, lung function, and exacerbation history (i.e. the standard care arm in this trial) are advised of any changes to their corticosteroid treatment on the day of their visit with a clinician, whereas a biomarker-based strategy introduces a delay to this clinical decision. To mimic this within the study, the personnel performing the study visits must therefore be unblinded regarding treatment group allocation. However, automated algorithms are used for both arms to produce treatment advice that is intended to remove any individual clinician bias. All subjects receive the same follow-up advisory phone call after the study visit to advise that the biomarker data are available and have advised a reduction, increase, or maintenance of current treatment to ensure subjects remain blinded to the intervention.

All centres within this study are being monitored by a Clinical Research Organisation (CRO) specialising in healthcare research delivery. Remote monitoring of data collection via the eCRF is also possible, permitting timely identification of incomplete or missing data and standardization of AE/SAE reporting. Furthermore all study co-ordinators have been provided with standardized worksheets reinforcing consistency in data collection.

Recruitment to date has been variable across sites for numerous reasons, i.e. differences in patient demographics and disease presentation, variability of medical record keeping and access to historical spirometry assessments, staffing issues, and patient engagement in other pharmacological studies. Strategies to promote and increase recruitment have been employed, e.g. weekly emails to all sites with tips for successful recruitment practice for more actively recruiting sites, continual CRO engagement with individual sites to troubleshoot difficult pre-screening scenarios, and coordinator teleconferences to allow direct interaction between all coordinators. In doing so, discrepancies regarding reasons for exclusion at the pre-screening stage have been identified and resolved.

## Trial status

Recruitment is currently active at all 12 sites. The first patient was randomised in January 2016. As of 13 October 2017, 222 patients have been randomised.

## Additional files

Additional file 1: Spirit checklist completed as per submission guidelines. (DOC 122 kb)

Additional file 2: Patient informed consent form template, plus additional Roche Clinical Repository consent form. (DOCX 101 kb)

## Abbreviations

ACQ-7: 7-item Asthma Control Questionnaire
AE: Adverse event
ANCOVA: Analysis of covariance
ANOVA: Analysis of variance
AQLQ: Asthma Quality of Life Questionnaire
CI: Chief Investigator
CONSORT: Consolidated Standards of Reporting Trials
CRO: Clinical Research Organisation
DNA: Deoxyribonucleic acid
e-CRF: Electronic case report form
FeNO: Fractional exhaled nitric oxide
FEV1: Forced expiratory volume in 1 second
FP: Fluticasone propionate
FVC: Forced vital capacity
GCP: Good Clinical Practice
GINA: Global Initiative for Asthma
ICS: Inhaled corticosteroid
IL: Interleukin
LABA: Long-acting beta agonist
NHS R&D: National Health Service Research & Development
OCS: Oral corticosteroid
PI: Principal Investigator
PRO: Patient-reported outcome
RCR: Roche Clinical Repository
RNA: Ribonucleic acid
SAE: Serious adverse event
SPIRIT: Standardized Protocol Items Recommendations for Interventional Trials
T2: Type 2
