# Supplementary material for: A randomised pragmatic trial of corticosteroid optimization in severe asthma using a composite biomarker algorithm to adjust corticosteroid dose versus standard care: study protocol for a randomised trial
Source: Trials. 2018 Jan 4;19:5. doi: 10.1186/s13063-017-2384-7 (PMC5753571; doi:10.1186/s13063-017-2384-7)
Supplement: Supplementary file 2 — Patient informed consent form template, plus additional Roche Clinical Repository consent form. (DOCX 101 kb) [file 13063_2017_2384_MOESM2_ESM.docx]

**Additional file 2:**

**Patient Informed Consent Form Template**

**(Version: 7.0 Date: 08 September 2015)**

| **Study title*A trial of adjusting steroid dosing in severe asthma using inflammation markers compared to asthma symptoms and lung function*** |
| --- |

**Principal Investigator’s name**

**Principal Investigator’s title**

**Telephone number of Principal Investigator:**

**Study nurse or coordinator’s name**

**Study nurse or coordinator’s contact number**

You are being invited to take part in a research study carried out at {*insert the group/organisation/university of the Principal Investigator (PI) or the PI’s name*}.

Before you decide whether or not you wish to take part, you should read the information provided below carefully and, if you wish, discuss it with your family, friends or GP (doctor). Take time to ask questions – do not feel rushed or under pressure to make a quick decision.

You should clearly understand the risks and benefits of taking part in this study so that you can make a decision that is right for you. This process is known as ‘Informed Consent’.

**You do not have to take part in this study and a decision not to take part will not affect your future medical care.**

You can change your mind about taking part in the study any time you like. Even if the study has started, you can still opt out. You do not have to give us a reason. **If you do opt out, it will not affect the quality of treatment you get in the future.**

Irrespective of your decision, we would like to thank you for your interest in this study.

| **Why are we doing this study?** |
| --- |

We are doing this study to find out if doses of steroids (both inhaled and oral), routinely prescribed can be better targeted in patients with severe asthma, without causing any worsening of asthma symptoms. High doses of steroids can lead to side-effects and it is better for the patient for their asthma to be controlled by as low a dose of steroids as possible.

It is thought that high doses of steroids do not improve all symptoms of asthma. It is not currently possible, however, to identify these patients easily in the doctor’s surgery. In this study, we hope to identify those patients who will benefit from higher doses of steroids and those who will not: this will be done by looking at markers of asthma in their blood and exhaled breath and by using these to optimise the steroid dose. In addition, identifying which symptoms and aspects of asthma do not respond well to steroid treatment may allow drug companies to develop new treatments for asthma.

This study is part of a larger programme of studies, called RASP-UK (Refractory Asthma Stratification Programme). This programme is being conducted by a group of clinical and academic experts from UK Universities and NHS Severe Asthma Centres, together with pharmaceutical companies with an interest in asthma.

| **Why am I being asked to take part?** |
| --- |

You are considered to have severe asthma as you take a number of inhaled medications plus occasional or regular oral corticosteroids and your doctor thinks you might be suitable for this study. Around 400 adults aged between 18 – 80 years will take part in the study in the UK.

**What happens if I don’t want to take part?**

It is up to you whether you decide to join the study. Your doctor will explain the study and go through this information sheet with you. If you agree to take part, they will then ask you to sign a consent form. You are free to withdraw at any time, without giving a reason. **Not taking part or deciding to withdraw later on will not affect the care you receive from your doctor.**

| **Who is organising and funding this study?** |
| --- |

This research study is being organised by a group of doctors (headed by Professor Liam Heaney at Queen's University Belfast) who are experts in asthma. It is supported by Asthma UK and is funded by the Medical Research Council (MRC), who will be responsible for providing payments to the study centre to cover the cost of staff and all study procedures.

Additional partners working on the study are several drug and medical device companies ( Aerocrine, Amgen, Medimmune, Roche/Genentech, Vitalograph) who are providing equipment, laboratory analysis or scientific expertise to the doctors involved.

| **How will the study be carried out?** |
| --- |

The study will take place at this clinic and at a number of other clinics in the UK. You will be in the study for a maximum of 12 months. You will be asked to come to the clinic for eight scheduled visits, and also to visit if you have had an worsening (exacerbation) of your asthma.

Four hundred people will take part in the study and it is expected that it will take 18 months for them all to be recruited.

The study has two stages:

1. Screening (up to 2 weeks)
2. A single-blind study period (up to 48 weeks). “Single-blind” means that your doctor will know what study group you are in, but that you will not.

If the results of your screening tests mean that you are suitable to continue in the study, you will be put into a study group for the ‘single-blind’ study period.

The study group you are put into is randomly selected, using a computer programme. This programme will decide if you are in the blood-and breath marker group or in the usual care group. At no point will your safety or care be compromised.

| Group | Study group | Chance of being in this group |
| --- | --- | --- |
| A | Blood and breath markers | 4 in 5 (80%) |
| B | Usual care | 1 in 5 (20%) |

**Study Assessments:**

The assessments that will take place at each stage are as follows:

**Screening:**

- Your doctor will review with you your past medical history and any treatments you are currently taking for asthma or other conditions
- You will have a physical examination which includes measuring your height, weight and vital signs (blood pressure, heart rate, temperature, oxygen saturation and breathing rate)
  - Oxygen saturation is a measure of how well the haemoglobin in your blood is carrying oxygen through your body. It is measured by a clip being placed on your finger for a minute or so)
- A breath test will measure Fractional Expiratory Nitric Oxide, (FeNO) a marker in your exhaled breath to see if there is a specific type of inflammation in your lungs
- Samples of your blood will be taken for routine tests to check your general health
- Some of these blood tests will be taken at each clinic visit and will measure blood cells, called eosinophils, neutrophils and periostin (a protein in the blood) which indicate the type of inflammation in your lungs. These tests will be used with the breath test (FeNO) to decide on the level of steroid treatment. Periostin measurement is not currently part of standard asthma care. The test for periostin is currently in development and is currently for investigational use only.
- Your blood or urine will also be tested to see if you have been smoking in the past 24 h. If you have been smoking you will not be eligible to take part in the study.
- If you are taking prednisolone, the concentration of that will be measured in your blood
- If you are taking theophylline, the concentration of that will be measured in your blood
- If you are a woman who could get pregnant a blood sample will be taken to check that you are not pregnant. Pregnant women cannot take part in the study.
- You will be asked to complete two questionnaires about your asthma and how it affects your daily activities
- You will have routine tests done to measure lung function called spirometry, testing the amount of air you can breathe out and the maximum speed at which you can do so
- A sample of sputum ( what you cough up from your lungs) will be taken
- A chest X-ray will be done if you have not had either a chest X-ray or chest CT scan during the past 24 months

Several of these assessments will be repeated during the study and these are shown in the table overleaf.

| Procedures | Screening visit | Baseline (Randomisation) | Visit 1 | Visit 2 | Visit 3 | Visit 4 | Visit 5 | Visit 6  Close-out visit |
| --- | --- | --- | --- | --- | --- | --- | --- | --- |
| **Time** | **-2 weeks**  **(±3 days)** | **0 weeks**  **(±3 days)** | **8 weeks**  **(±3 days)** | **16 weeks**  **(±3 days)** | **24 weeks**  **(±3 days)** | **32 weeks**  **(±3 days)** | **40 weeks**  **(±3 days)** | **48 weeks**  **(±3 days)** |
| Informed consent | X |  |  |  |  |  |  |  |
| Medical history | X |  |  |  |  |  |  |  |
| Temp, BP, Pulse, oxygen saturation and respiration | X |  |  |  |  |  |  |  |
| Weight & Height including BMI | X |  |  |  |  |  |  | X |
| Physical examination | X |  |  |  |  |  |  |  |
| Pregnancy test (where relevant) | X |  |  |  |  |  |  |  |
| Asthma control Questionnaire | X | X | X | X | X | X | X | X |
| Asthma Quality of Life questionnaire |  | X |  |  |  |  |  | X |
| Spirometry | X | X | X | X | X | X | X | X |
| Serum or urinary cotinine | X |  |  |  |  |  |  |  |
| Serum periostin  (Central lab) | X | X | X | X | X | X | X | X |
| Haematology - blood eosinophil and neutrophil count | X | X | X | X | X | X | X | X |
| Serum biochemistry and theophylline (if on theophylline) | X |  |  |  |  |  |  |  |
| Serum prednisolone / cortisol (if on prednisolone) | X |  |  |  |  |  |  |  |
| Fractional Expiratory Nitric Oxide ( FeNO) | X | X | X | X | X | X | X | X |
| Urine sample | X | X |  |  | X |  |  | X |
| Plasma, serum and Paxgene sample for biobanking |  | X |  |  | X |  |  | X |
| Optional DNA sample for biobanking |  | X |  |  |  |  |  |  |
| Induced Sputum |  | X |  |  |  |  |  |  |
| Information collected on exacerbations and associated treatment | X | X | X | X | X | X | X | X |
| Review/reporting of illnesses and side effects ( adverse events) | X | X | X | X | X | X | X | X |
| Review of self-management plan inhaler technique and medication adherence (on study) | X | X | X | X | X | X | X | X |
| Follow-up telephone call (or similar) |  | X | X | X | X | X | X |  |
| Handover to routine care (End of Trial) including question on “Which study arm “ |  |  |  |  |  |  |  | X |

After each study visit, the study doctor will look at the results of your blood and breath tests and will decide if you need to adjust the dose of your inhaled steroid. The study nurse will phone you within 3-7 days after the clinic visit to tell you which dose of inhaled steroid to take from then until the next scheduled clinic visit. It is very important that you adhere to this dose until your next clinic visit, unless you have an exacerbation of your asthma.

The additional tests done during the study are:

- - Samples of sputum taken for measurement of markers of asthma
  - Blood samples taken for future measurement of markers of asthma

These samples will be stored for future research on asthma and other respiratory disease

**What happens if my asthma gets worse between routine study visits to the clinic?**

If your asthma gets worse before you are due to return to the clinic, we will ask you to follow your usual self-management plan. We will ask you to contact the study team to arrange to come to the clinic within 72 h of the worsening. If that is not possible, for example if you are in hospital, please contact the study team to arrange to come to the clinic as soon as you can

At those visits the following will be done:

- A note taken of the date when your asthma got worse
- A note taken of how you have adjusted your asthma treatment
- Your vital signs (blood pressure, heart rate, temperature, oxygen saturation and breathing rate) will be measured
- You will be asked to complete a questionnaire about your asthma symptoms
- Spirometry
- Measurement of FeNO
- Blood sample taken for measurement of signs of inflammation and for measurement of gene expression
- Urine sample taken for measurement of signs of inflammation
- Sputum sample taken, if you can cough up sputum, for measurement of markers of worsening asthma

**If you have to attend any hospital appointments, other than those related to the study, while you are taking part, please let the hospital staff know that you are a participant in this study.**

**What will happen to the samples I give?**

Blood, sputum and urine samples

Blood samples will be collected for routine tests (including blood chemistry and blood counts), to determine the amount of periostin in your blood and for future research on substances related to your asthma or other respiratory diseases. Sputum will be analysed to look at markers of inflammation and infection. Urine will be used for pregnancy tests (if you could become pregnant), to assess if you have been smoking and to measure markers that may indicate the severity of your asthma. Analysis of these markers is not part of standard practise.

The total volume of blood that will be collected from you during the whole study is approximately 20 tablespoons (305 mL), including .5 tablespoons (tbsps.) (75 mL) during the screening period. At two other visits during the study, 4.3 tbsps (65mL) will be collected. At each of the other five clinic visits, a maximum of 1.3tbsps (20mL) will be collected. In total, this is less blood than you would give during a standard blood donation.

These tests are done to ensure your safety and to allow doctors to measure what is happening due to changes in your asthma medication. These samples will be stored until the study results have been reported, with the exception of blood and urine samples for substances related to your asthma, which will be stored for a maximum of 15 years after all study data have been collected; these blood samples may be used in the future by the doctors and companies involved in the study for research relating to asthma. The data resulting from the stored samples will be kept confidential as described in the section ‘is this study confidential’.

Genetic samples (DNA only)

A separate blood sample for genetic testing will be taken but only if you give your additional permission for this to be done.

If you give consent for this sample, you will be agreeing to your anonymised information and blood sample being processed by Roche, one of the pharmaceutical companies working with the academic doctors. You will be agreeing to have anonymised information passed to other Roche group companies, to companies working with Roche, and to health authorities. Some of these may be in countries outside the UK.

Roche or its other group companies may use your anonymised information and sample for future medical research in areas other than asthma.

***Please see Part 2 of this Information Sheet for further details. Taking part in this section of the study is optional.***

| **What will happen to me if I agree to take part?** |
| --- |

During the study you will see your usual doctors and nurses at clinic visits. Your medical records will be viewed by representatives of the company managing the study on behalf of the doctors running the study. All of these individuals have signed relevant confidentiality agreements.  Any data removed from the hospital will only be identified by a randomisation number and will not be able to be identified as related to you.

During the study you should:

- Please attend all your study appointments. If you know that you will miss an appointment, contact the study staff to reschedule it as soon as possible
- Take your morning dose of asthma medications before attending for your study appointment
- Follow your asthma management plan if you have a worsening of your asthma
- Tell the study staff about any side effects, GP or hospital visits that you may have, as well as any medicines or supplements you might be taking
- Tell the study staff if you believe you or your partner might be pregnant. Use contraception as directed by the study staff
- Ask questions as you think of them
- Tell the study staff if you change your mind about staying in the study

During the study you should not:

- Take part in any other drug studies. This is to protect you from giving too many blood samples

| **What other treatments are available to me?** |
| --- |

If you decide not to take part in the study, your doctor will continue to treat your asthma in accordance with best practice and international asthma management guidelines.

| **What are the benefits?** |
| --- |

There is no guarantee that you will receive any benefit from this study, and taking part in this study may or may not cause your asthma to improve. Information from this study may help doctors learn more about asthma and how to adjust your and other people’s doses of steroids more effectively.

| **What are the risks?** |
| --- |

There are possible risks, disadvantages and inconveniences with any research study. Consider these carefully before agreeing to take part in this study.

**Tests and Procedures**

You will potentially have more tests and procedures if you take part in the study, compared to standard hospital visits. Study visits could take more time than standard hospital visits and you are likely to have more blood taken.

**Blood tests**

During this study, small amounts of blood will be drawn from a vein, usually in your arm, and used for tests that allow the study doctor to see how you are doing. Drawing blood may cause pain where the needle is inserted, and there is a small risk of bruising or infection at this location. Some people experience dizziness, upset stomach, or fainting when their blood is drawn. The study doctor or study staff may apply a topical cream to numb your skin to decrease the pain where the needle is inserted before drawing blood.

**Scans and radiation**

If an x-ray or CT scan of your chest from the previous 24 months is not available for your doctor to review, you will receive a chest x-ray during the screening period. This chest x-ray is additional to what you would normally receive as part of your care. During the chest x-ray you will be exposed to a small amount of radiation, which is equal to the same amount of radiation the average person receives in 3 days just from their surroundings.

Exposure to radiation has an associated risk of developing cancer in later life. For the level of radiation used in this study, the risk of this is very small being approximately a 1 in 1 million additional chance of developing cancer. For comparison, the natural risk of developing cancer is approximately 1 in 3.

**Insurance**

Before taking part you should consider if this will affect any insurance you have, including travel insurance, and seek advice if necessary.

| **What if something goes wrong when I’m taking part in this study?** |
| --- |

If you have a concern about any aspect of this study, you should ask to see the study staff who will do their best to answer your questions.

If you remain unhappy and wish to complain formally, you can do this through the NHS Complaints Procedure. Additional information is available from your local Patients Advice and Liaison Service Office [insert local details].

In the event that something does go wrong and you are harmed during the research and this is due to someone’s carelessness, then you may have grounds for a legal action and compensation against Queen’s University Belfast but you may have to pay your legal costs.

| **Will it cost me anything to take part?** |
| --- |

You will receive payment for your travelling expenses for clinic visits that are additional to your usual schedule of clinic visits.

| **Will I be paid for taking part in the study?** |
| --- |

There is no payment for, or compensation for inconvenience of, taking part in the study.

| **Is the study confidential?** |
| --- |

As your GP is a critical member of the medical team looking after you, they will be told that you are taking part in the study.

Once on the trial you will be identified only by a unique code number and information about the code will be kept in a secure location and access limited to research study staff at your hospital. The data will be coded, stored and protected by the study Sponsor for 15 years after the end of the study.

Your data, including trial records, information about your general health, how you have responded to the changes to your asthma medication, any side effects that you may have experienced and the results of any tests carried out during the study, will be collected by your doctor and provided to the team of doctors running the study and the other companies involved. All of these individuals have signed relevant confidentiality agreements.  Any data removed from the hospital will only be identified by a randomisation number and will not be able to be identified as related to you

Your data will be analysed by the team of study doctors and the companies acting on behalf of them to work out whether the changes to asthma medication have caused any change to the symptoms of the people taking part in the study. Your data may be analysed outside the UK. Certain statistical tests will be carried out on your data, along with that collected from the other participants who entered the study.

The study doctors may then forward the results of the study to government bodies worldwide, responsible for health policy, and the results may also be used in reports of the study or for scientific presentations or publications. No personal information will be included in these presentations and publications and you will not be identified in them.

Depending on the results of the study, the study doctors may need to re-analyse the data from this study at a later date. They may need to carry out extra tests on samples collected during the study or perform further statistical tests on the data. The results of this study may be used for future medical research

At any time during or after the study, staff from the study sites, companies acting on behalf of the study doctors, health authorities, such as the Medicines and Healthcare products Regulatory Agency (MHRA) and Trust Research and Development departments will occasionally be granted direct access to your medical records, so that they can confirm that the information collected during the study is accurate. In these circumstances your identity may be disclosed but will remain absolutely confidential.

The results of the research will be published in a report and shared with the other research doctors on the study and shown to other doctors at meetings. The results of the trial will eventually be posted on <http://www.ClinicalTrials.gov>, approximately one year after the end of the trial. You will not be identified in any report or publication.

| **Where can I get further information?** |
| --- |

If you have any questions about this research study, the procedures, risks or benefits or alternative treatments, please call the study staff using the information on the front page of this booklet.

You can also discuss your participation with your regular doctor.

If you have any questions about your rights as a patient in this study you may want to contact your local Patients Advice and Liaison Service Office *[insert local details].*

You will be given a copy of this information sheet and a copy of your signed consent form to keep. You should keep this information in a safe place and in your possession for as long as you are in the study.

During the study, if there is an emergency please contact your doctor on the telephone number given. Should you have to visit another doctor tell him/her that you are taking part in this study so that he/she can contact your study doctor if necessary.

You can find general information about clinical trials at the following websites:

[www.nhs.uk/Conditions/Clinical-trials](http://www.nhs.uk/Conditions/Clinical-trials)

[www.peopleinresearch.org](http://www.peopleinresearch.org)

[www.healthtalkonline.org/medical_research/clinical_trials](http://www.healthtalkonline.org/medical_research/clinical_trials)

If you need any further information now or at any time in the future, please contact:

Name

Address

Phone No

***Part 2***

**ROCHE CLINICAL REPOSITORY**

| **Introduction** |
| --- |

The Investigators want to better understand why certain patients are more likely to respond to treatment than others, to facilitate the development of personalized medicines - to get the right medicine to the right patient. They would like to store DNA taken from a blood sample for genetic analysis for research .If you agree to donate a DNA sample for research; it will be stored in the Roche Clinical Repository (RCR), a place where human samples are securely stored. Your samples will be stored in the RCR for up to 15 years.

Taking part in the RCR is entirely voluntary. You do **not** have to donate samples to the RCR. You will get the same care, whether you donate samples or not. You also can decide to donate now, and then change your mind later. No matter what you decide, it will not affect your participation in the main study or your medical care.

| **Why are these samples being collected?** |
| --- |

The samples in the RCR may be used to help researchers to:

- Better understand why certain people are more likely to respond to medicines such as steroids
- Better understand how and why asthma, asthma-related diseases, or other respiratory diseases act differently in different people
- Develop new treatments for asthma, asthma-related diseases, or other respiratory diseases
- Find reasons why certain people are more likely to have side effects to medicines such as steroids
- Develop better ways for preventing diseases or treating diseases earlier
- Develop or improve tests that help with detection or understanding of asthma and related diseases to help identify the right medicine for the right patient

Results from this research will be based on information from all of the patients who contribute samples.

| **What will happen if I donate a sample?** |
| --- |

If you agree to donate the DNA sample to the RCR, the sample will be collected at the same time as one of your scheduled blood draws, so you will not have to undergo any additional procedures. This will include the following sample:

- An additional blood sample of 7.5 mL (approximately 1/2 tablespoon) for genetic testing (to study what information is inherited)

| **What are the possible side effects or risks?** |
| --- |

The risks associated with drawing blood are described earlier in this consent form (see ‘Possible Risks and Discomfort Associated with Drawing Blood’).

| **Are there benefits to donating samples?** |
| --- |

You will not receive any direct benefit from donating your DNA sample. However, research performed on this sample may benefit other patients with asthma or a similar condition in the future.

| **Will I be paid if I donate samples?** |
| --- |

You will not be paid for donating a DNA sample to the RCR.

Information from this research may lead to discoveries and inventions or development of a commercial product. The rights to these will belong to Roche. You and your family will not receive any financial benefits or compensation from or have rights in any developments, inventions, or other discoveries that might come from this information.

| **How will my privacy be protected?** |
| --- |

Roche will use the collected DNA samples for research about genetic (inherited) factors. To ensure that your health information is kept confidential, your blood sample and health information will be labeled with your unique patient identification number when it arrives at Roche. Roche will replace your patient identification number with a new number. The link between your patient identification number and the new number will be stored safely in a database that is available only to authorized individuals. This means there is an extra layer of privacy for the DNA samples (see the picture below).

| You learn about RCR sampling when you enter the study | You read and sign this form | The blood sample for genetic analysis and your health information are given a unique patient identification number | At Roche, the original patient identification number is replaced with a new number | The link between the original number and the new number is safely stored, and the sample may only be linked back to you by very few designated people following strict procedures | RCR genetic research is performed on confidential health information about you |
| --- | --- | --- | --- | --- | --- |


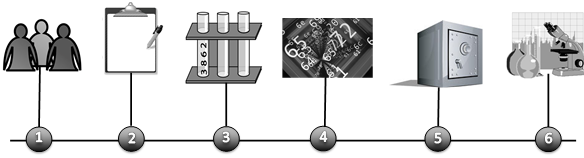


Although this genetic research will be done in a manner that does not identify you, it may be necessary to link the new number assigned to your samples and health information back to your patient identification number. This will be done only if it is necessary to find your sample when the storage period is over, if you change your mind about storing samples in the RCR, if the research results need to be part of the information sent to health authorities, or safety reasons. Your samples and health information will be linked back only to your patient identification number, never to your name.

Information from the sample analysis will not be made available to you or to your doctor, unless required by law. Information from the analysis will not be part of your medical record and will not be given to your insurance company or employer.

Investigators from the RASP study, drug and device companies and companies who partner with RASP may study the RCR samples and associated research data in any country worldwide. Roche may then send the research results to health authorities worldwide. Information from this research may be published in a medical journal or presented at scientific meetings, so that other doctors can find out about the results. **Your identity will not be disclosed**. You will not be identified in any study reports or presentations

| **May I change my mind about storing my samples in the RCR?** |
| --- |

Yes. You may change your mind at any time. If you want to withdraw your consent for the use of your DNA sample during the study, tell your study doctor that you no longer want your DNA sample stored or used for research. If you want to withdraw your consent after the close of the study, follow the instructions provided to you by the study doctor. Then, any samples that remain will be destroyed. If you change your mind and your DNA sample has already been tested, those results will still remain as part of the overall research data. In the event of your death or loss of competence, your specimens and data will continue to be used as part of the RCR. If you withdraw or discontinue from the main study, your RCR samples will continue to be stored and used for research unless you specifically ask that they be destroyed.

| **Who do I contact if I have questions?** |
| --- |

You can call Dr. {Study Doctor} at {telephone number} at any time if you have questions about the RCR or if you think you have experienced a research-related injury.

If you have any questions about your rights as a patient in this study you may want to contact your local Patients Advice and Liaison Service Office *[insert local details].*

**OPTIONAL ROCHE CLINICAL REPOSITORY SAMPLING**

**Consent Form**

I have read the information given to me. I understand what the Roche Clinical Repository (RCR) is for. I also understand any potential risks and benefits of donating a blood sample from which DNA will be taken.

**□ I do not want to donate a DNA sample. I do not want to take part in the optional RCR study.**

**[If you don’t want to participate, please tick this box only. You do not need to sign anything.]**

or

**□** I **do** want to donate a DNA sample to the RCR.

I give my consent to the doctor collecting and processing my samples and information about my health in the study titled, a pragmatic trial of corticosteroid optimisation in severe asthma using a composite biomarker algorithm

I consent to my information and blood sample being processed by Roche. I also consent to have my information passed to other Roche group companies, to companies working with Roche, and to health authorities. I understand that some of these may be in different countries*.*

I give my consent to Roche or its other group companies to use my information and sample for future medical research. This includes my health information collected in this study.

I have had a chance to ask questions. All of my questions have been answered. I understand that I will be given a copy of this consent form after signing it.

If I withdraw my consent for my DNA sample to be stored in the Biobank, I understand my sample will be destroyed. But I also understand that Roche and others may still use any information already collected about me up until the time I withdraw my consent.

|  |  |  |
| --- | --- | --- |
| Print Name | Participant’s Signature | Date of Signature |

*(Participant to write own name) (Participant to personally date)*

I, the undersigned, have fully explained the relevant details of the storage of a specimen in the RCR to the participant named above; I will ensure the participant receives a copy of their signed consent.

|  |  |  |
| --- | --- | --- |
| Print Name | Investigator/Designee Signature | Date of Signature |

*(Investigator/designee to write own name) (Investigator/ designee to personally date)*
